# Additive Manufacturing of Large Coreless Filament Wound Composite Elements for Building Construction

**DOI:** 10.1089/3dp.2020.0346

**Published:** 2022-06-09

**Authors:** Serban Bodea, Pascal Mindermann, Götz T. Gresser, Achim Menges

**Affiliations:** ^1^ Institute for Computational Design and Construction (ICD), University of Stuttgart, Stuttgart, Germany.; ^2^ Institute for Textile and Fiber Technologies (ITFT), University of Stuttgart, Stuttgart, Germany.; ^3^ German Institutes of Textile and Fiber Research (DITF), Denkendorf, Germany.

**Keywords:** additive manufacturing, robotic coreless filament winding, fiber-reinforced polymers, fiber tension control, robotic fabrication, cyber-physical production system, robotic motion-control, automated construction

## Abstract

Digitization and automation are essential tools to increase productivity and close significant added-value deficits in the building industry. Additive manufacturing (AM) is a process that promises to impact all aspects of building construction profoundly. Of special interest in AM is an in-depth understanding of material systems based on their isotropic or anisotropic properties. The presented research focuses on fiber-reinforced polymers, with anisotropic mechanical properties ideally suited for AM applications that include tailored structural reinforcement. This article presents a cyber-physical manufacturing process that enhances existing robotic coreless Filament Winding (FW) methods for glass and carbon fiber-reinforced polymers. Our main contribution is the complete characterization of a feedback-based, sensor-informed application for process monitoring and fabrication data acquisition and analysis. The proposed AM method is verified through the fabrication of a large-scale demonstrator. The main finding is that implementing AM in construction through cyber-physical robotic coreless FW leads to more autonomous prefabrication processes and unlocks upscaling potential. Overall, we conclude that material-system-aware communication and control are essential for the efficient automation and design of fiber-reinforced polymers in future construction.


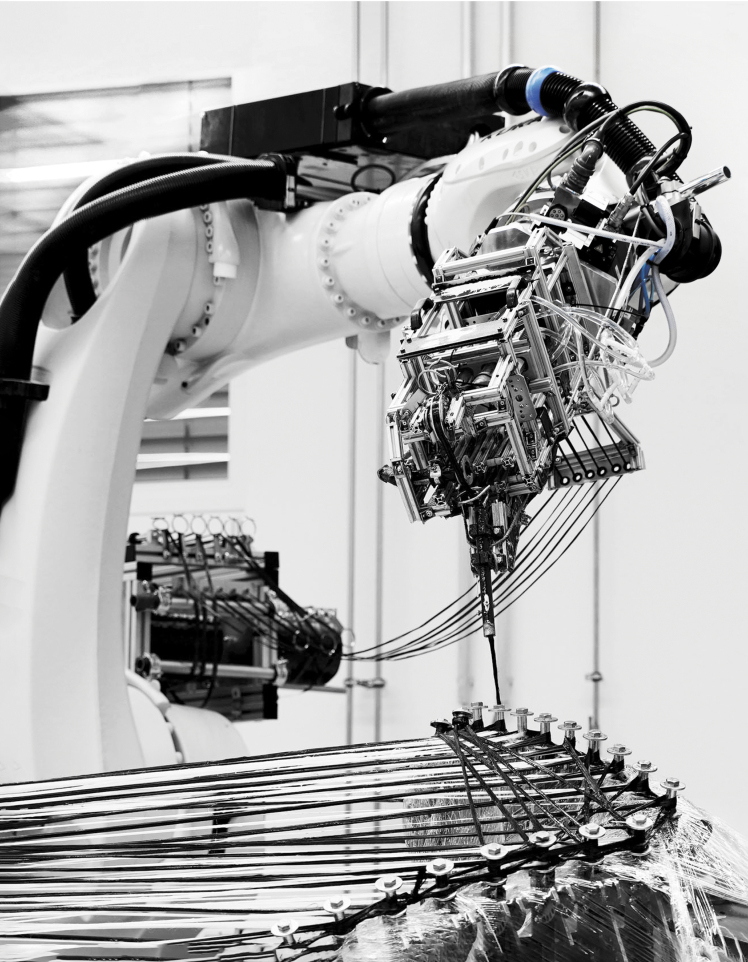


## Introduction

The building industry represents 15% of global GDP^1^ and is a leading employment sector,^2^ yet it is one of the least digitized^3^ and least productive^2^ industrial sectors. Construction must close a productivity gap estimated in 2017 at 1.63 trillion dollars^2^ and add more value to its core societal role. Automation and digitization can be leveraged for increased productivity. Digital fabrication research tackles these challenges while simultaneously addressing the rising demand for material-efficient construction through customized manufacturing.^4^

Additive manufacturing (AM) shifts the paradigm from mass-production to mass-customization in construction. The AM uses materials characterized by isotropic (concrete,^5–7^ unreinforced plastics,^8^ and metals^9,10^) or anisotropic (fiber-reinforced polymers [FRPs] with thermoplastic^11,12^ or thermoset matrices^13^) material properties. These applications profit from material science and industrial robotics advances, adapting their AM methods to construction-specific needs.^14^

The use of specific material systems must be application- and fabrication-process-aware. Long-span construction, for instance, demands lightweight, high-strength, and highly formable materials to achieve stiffness-through-form. The FRPs are materials that exhibit excellent strength-to-weight ratios under various loading conditions, making them ideal for such structures. Moreover, FRPs are well suited for AM processes as they inherently include reinforcement. For example, Branch Technology has demonstrated a robotic three-dimensional (3D) printing technology that solidifies a mixture of acrylonitrile butadiene styrene and carbon fiber (CF) that is able to create 3D-printed space-frames such as the One City Pavilion.^15,16^ The CF 3D printing applications such those proposed by Kwon *et al.*^11,12^ have offered evidence of CF added to 3D-printed structures as reinforcement. Both technologies are examples of customization but are slow and utilize thermoplastics, which may pose construction limitations. Although Branch Technology methods were verified at pavilion scale, their homolog at ETH is yet to be proven scalable.

The AM technology with the potential to integrate tailored reinforcement at a production speed suitable for construction is Filament Winding (FW) and its variant Coreless Filament Winding (CFW). The CFW drastically reduces the need for molds or mandrels, an advantage that makes it well suited for largescale and bespoke applications.

At the University of Stuttgart, the integration of industrial robots into CFW processes for technical fiber systems, glass fibers (GF), and CF composes the Robotic Coreless Filament Winding (RCFW) research stream.^17,18^ The ongoing research yields novel industrialized production models exhibiting various automation, scalability, and efficiency.^13,17–22^

Continuing this line of research, we investigate material-aware automation strategies for RCFW adapted to the structural and functional needs of lightweight construction. The challenge to develop smarter RCFW construction methods extends our research scope beyond file-to-factory application, into the field of cyber-physical systems (CPS).^23^

## State of the Art

### Cyber-physical systems

The CPS are open systems of collaborating cyber-physical entities linked into data acquisition, processing, and sharing via information networks^23^; they are key enabler-technologies for “Industry 4.0,”^23–25^ the currently dominant industrial automation and data exchange paradigm. Their performance indicators are process stability, performance, reliability, and robustness, all of which are key to developing engineered systems integrating computation, communication, and control.^23,24^ A comprehensive literature review on CPS is available from Wu *et al.*^25^ whereas the terminology's development,^23^ and evolution,^26^ are described by Monostori *et al.*, Kim *et al.*, and Wu *et al.*, respectively.

The RCFW is an example of the sinuous development that many emerging technologies undergo, from initial conceptualization to large-scale construction. As pointed out by Monostori *et al.*^23^ and Hack *et al.*,^27^ research and development of construction-adapted CPS depend on information technology and market pressures and advances in manufacturing.^28^ The RCFW is no exception. In reviewing the progress in the field contextualized by research conducted at the University of Stuttgart, Vasey and Menges^29^ argue that the full potential of CPS in Architecture Engineering and Construction has not yet been reached. The authors provide evidence of the innovation and education interplay for developing CPS as part of academic research.

### Toward construction-ready RCFW CPS

Recent research has sought to develop manufacturing methods and verify them at building scale, utilizing technology transfer from the composite industry to construction. Interesting for our work are several academia^13,17,19^ and industry^30–32^ applications that have adopted CFW to reduce the need for formwork in AM construction elements. With the reduction of formwork come limitations in the types of producible structures. Because it is dependent on the incremental deformation of free spanning fibers, CFW is currently limited to the production of lattices that approximate anticlastic surfaces,^19^ 3D frames,^32^ or truncated cone tubes.^30^

With the exception of processes described by Dawson^30^ and Minsch *et al.*,^31^ CFW methods still do not provide complete design, analysis, simulation, and fabrication solutions that are adaptable for construction.

Nevertheless, digital fabrication methods enable new architectural applications for composite material systems. The ICD/ITKE Research Pavilion 2013–14 was a collaborative robotics application that utilized two synchronized industrial arms to prefabricate Glass/Carbon Fiber Reinforced Polymers (G/CFRP) building components.^18^ Automated process monitoring and quality control were outside its scope. This research laid the ground work for many of the future RCFW applications.^13,19,29,33^ The ICD/ITKE Research Pavilion 2014–2015 developed the first CPS for tape laying preimpregnated CF tows on an inflatable ETFE membrane. Here, the position of the end-effector on the membrane was controlled through feedback with the industrial robot.^33^ Its advances in robot control were partially utilized for distributed robotic manufacturing processes demonstrated by the ICD/ITKE Research Demonstrator 2016–2017 with a high degree of automation and coordination of the collaborating robotic agents: two industrial robots and a drone for transferring the fiber.^34^

The precursor to our application was an RCFW method to fabricate elongated tubular composite elements.^35^ The technology, developed at the University of Stuttgart and upscaled in collaboration with FibR GmbH, was verified in the prefabrication of the BUGA Fibre Pavilion's load-bearing structure.

### Research gap

An analysis of state of the art reveals that all RCFW applications are interdisciplinary. They also span *in-situ*^33^ and prefabrication^30,35^ embodiments. Regarding CFW prefabrication, it is revealed that solutions are project-specific. This specificity has a limitative effect on RCFW control methods, tools, and solutions. The presented application, named Cyber-Physical Robotic Coreless Filament Winding (CPRCFW), aims at addressing a need for generality, versatility, and reusability of methods and tools demanded by novel building systems such as the BUGA Pavilion.^35^ This research gap will be addressed through more general and extendable control methods, modular software and hardware tools, and clearly defined automation protocols. These principles will be embodied by a CPS consisting of feedback-driven, sensor-guided tension control and fiber impregnation methods embedded in an RCFW process. In addition, the application will address the upscaling potential of the CPRCFW methods and C/GFRP building structures as a direct result of the senor-informed fabrication method.

## Materials and Methods

**FIG. 1.**
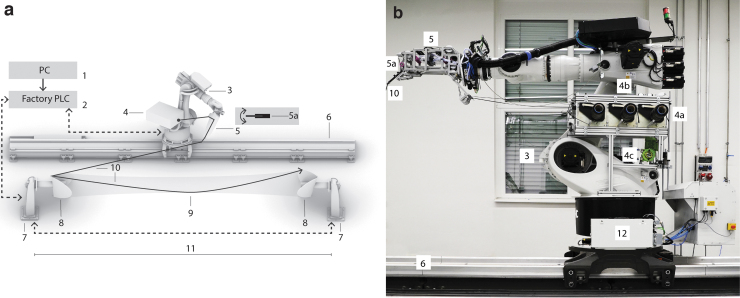
CPRCFW system: **(a)** conceptual diagram; **(b)** implemented system. Components: 1. Computer; 2. ICD fabrication laboratory PLC; 3. Industrial robot (KR420); 4. Fiber guiding and impregnation system: 4a. CF/GF fiber creel, 4b. passive tensioning system (mechanical dancer bar), 4c. Peristaltic pump: Albin ALP 09-F connected to Polycarboxylic epoxy resin source; 5. Fiber impregnation end-effector; 5a. Tension sensor (Tensometric M-1191-KA); 6. Linear track, length 10 m; 7. Digitally synchronized 1-axis positioners (KP1), no core or mechanical synchronization needed; 8. Modular winding effectors, steel, weight 75 kg; 9. Multi-material G/CFRP composite; 10. Fiber bundle under pretension; fiber bundle on the composite body; 11. Adjustable distance between winding tools allows the AM of any component length in the 1 to 10-m range; 12. BEC Box: digital/analog sensors and actuators integration unit. CF, carbon fiber; CPRCFW, Cyber-Physical Robotic Coreless Filament Winding; G/CFRP, Glass/Carbon Fiber Reinforced Polymer; GF, glass fibers; PLC, Programmable Logic Controller.

Owing to upscaling and digital control and monitoring, a re-characterization of the material system, fiber impregnation systems, and the kinematic system is required. The proposed system is pictured in Figure 1. Generally, the industry assesses that higher initial design effort and research investment in composite AM yields higher structural performance than is achievable by any individual component.^36,37^ Thus, we expect similar returns in construction applications in similar R&D conditions. In describing the research methods, results and evaluations of the fiber tension will be interchangeably given in units of force (N) or equivalent mass (kg).

### Properties and specifications for a CFW-adapted material system

A prerequisite for FRP applications throughout the industry is the development of lightweight materials that combine enhanced stiffness with high strength and toughness.^38,39^ Grossman *et al.* explain that most synthetic materials cannot combine high strength with increased toughness, because the constituent chemical bonds cannot resist and facilitate stress-induced deformation.^40^ The authors conclude that “gains in toughness are normally accompanied by a reduction in strength and vice versa.” However, this shortcoming can be mitigated through hierarchical composite architectures, as seen in many natural materials.^40^

We formulate a similar research question for composite manufacturing processes in construction: We need to reconcile seemingly contradictory demands, for high strength and toughness, through hierarchical composite architectures achievable through AM processes. It is well known that CF and GF materials have excellent mechanical properties.^38^ Moreover, at fiber volume ratios of 35–50% the performance of the FRP significantly exceeds the performance of its constituent elements.^39^ Further, owing to mechanical anisotropy, fibers can be engineered and precisely placed for high structural performance through FW^36,41^ and CFW. The second major factor influencing performance is form. For example, Vasiliev *et al.* illustrate the structural characteristics of anisotropic grids and the interrelation between overall form and local structural properties.^42,43^ In G/CFRP, fibers take tension, whereas the polymer matrix is mainly active in compression, distributing the force flow.^44^ For our own work, the mechanical properties of the RCFW elongated fiber lattices are evaluated in Gil Pérez *et al.*^45^ and for the specific application in domes composed of tubular elements, in Rongen *et al*.^46^ At component and structural systems scale, safety factors have been adapted to the multiple load cases that determine the application.^47^

A distinct advantage of manufacturing through CFW compared with working with two-dimensional woven textiles^44^ is the capacity to place every fiber bundle individually. In addition, the system retains its mechanical flexibility during fabrication because the open time of the thermoset resin can be precisely controlled. Tailoring the fiber orientation is essential: Even minor variations may lead to significant mechanical performance deviations.^48^ An analysis of the impact of fiber morphology on robotically wound test specimens that reports excellent structural performance under axial compression and axial tension is presented in Gil-Pérez *et al*.^49^

#### Fiber system

A hybrid six-roving GF and CF system was selected for the application. The number of utilized fiber-rovings was influenced by the upscaled fabrication system. Roving sizing was correlated to impregnation-cartridge volume, as explained in Mindermann *et al*.^50^ and as shown in Table 1.

**Table 1.** Fiber System: Material Properties of Glass Fibers and Carbon Fibers

Material	Product	Tensile modulus (GPa)	Tensile strength (MPa)	Elongation at break (%)
CF	Teijin Tenax-E STS40 F13 48K 3200tex	250	4300	1.7
GF	Owens Corning PipeStrand S2300 2400tex LS BP11 S CF A	81	3750	4.9

CF, carbon fiber; GF, glass fibers.

Only CF reinforcement is considered load-bearing since Young's modulus (250 GPa/81 GPa ≈309%) and the tensile strength (4300 MPa/3750 MPa ≈115%) of CF are higher compared with those of GF, which are therefore used as an integrated elastic mold.

#### Matrix system

The chosen polycarboxylic^51^ system (Table 2) consists of a resin sourced from renewable resources with an unlimited open time at 20°C and a viscosity of 450 MPa*s premixed with an activator. The low viscosity of the thermoset resin system was informed by emerging constraints arising from the fabrication method and setup upscale. This translated to:

**Table 2.** Matrix System: Material Properties of the PTP Resin (96 wt% Premixed Resin/Hardener, 4 wt% Accelerator)

Density (g/cm^3^)	Viscosity (MPa^*^s)	Flexural modulus (GPa)	Flexural strength (MPa)	Elongation at break (%)	Glass transition temperature (°C)	Pot life at 20° C
1.075	450	2.1	80	3	115	∞

- longer cycle times owing to larger components
- longer open times for the thermoset epoxy resin matrix

### System for fiber guiding, tension measurement, and fiber impregnation

The implementation of the CPRCFW system consisted of two interlinked steps. First, sensing and evaluation methods for fiber tension were integrated into the RCFW procedure. Second, automatic, in-line fiber impregnation was added, to achieve a 50% fiber/volume ratio. The GF, CF, and epoxy matrix were housed on the robot arm, in a bespoke compact configuration consisting of:

- a two-row CF creel with a capacity of six textile bobbins (spool holders: TC200-14-110^52^; capacity max. 10 kg each; integrated adjustable brake), in a modular metallic construction attached to robot-axis 1 (Fig. 1b:4a).
- a passive tension-control mechanism (mechanical dancer-bar^53^ with a 1.2-m stroke) housed above the CF fiber creel, consisting of six pulleys and adjustable counterbalance weight (Fig. 1b:4b).
- The decentralized fiber impregnation system consisted of two main components:
- an industrial peristaltic pump (type: Albin ALP 09-F; capacity: 27–70l/h; max. pressure: 2 bar) mounted underneath the creel (Fig. 1b:4c), supplied with premixed resin through a 10-mm diameter glass-fiber-reinforced hose^54^
- a bespoke robotic end-effector^50^

**FIG. 2.**
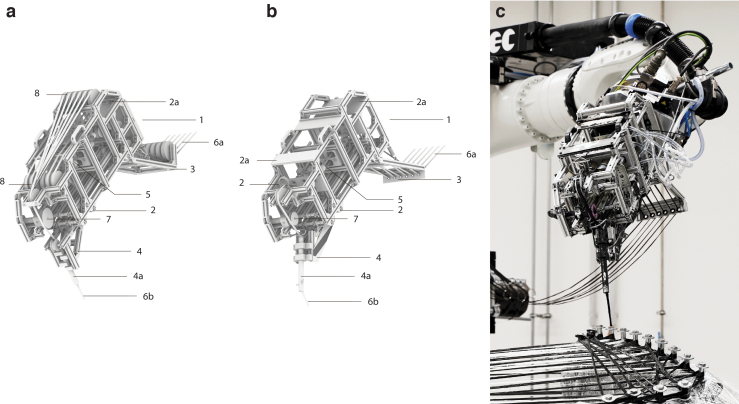
Robotic fiber impregnation end-effector: **(a)** Development iteration 1—features a complex fiber routing subsystem, with multiple ceramic rollers to change fiber direction; **(b)** Development iteration 2—a simplified, more robust version, features stiffening plates, a redesigned TCP assembly and simpler fiber routing; **(c)**. Robotic end-effector, in operation: CF CPRCFW; Components: 1. Robot flange interface; 2. Modular aluminum-profile/ring steel guide structure; 3. On-board tension control arm; 4. TCP assembly; 5. Fiber impregnation cartridges; 6a. Dry, individually routed fiber tows; 6b. Impregnated, assembled fiber tows; 7. Tension sensor; 8. Ceramic roller guides. TCP, Tool Center Point.

The modular robotic end-effector served as a research platform. Its development was completed in two iterations (Fig. 2a and b). The device's structure, attached to robot-axis six, consisted of an aluminum frame stiffened with planar elements to withstand multidirectional dynamic loading of up to 600 N (∼60 kg), see force distribution for GF and CF. The end-effector performs four integrated functions:

- GF and CF fiber guiding from the fiber creel to the impregnation cartridges^50^
- impregnation of GF and CF fiber rovings with automatically dosed epoxy resin
- measurement of tension on the impregnated fiber roving
- guiding of fiber rovings around winding pins

Dry fiber rovings are separately routed to the impregnation cartridges connected through branching tubes to the resin pump (i–ii). After impregnation, the rovings are assembled in a single bundle before reaching the sensor roller where the fiber tension (iii) is measured (Fig. 2b). A sub-ensemble of mechanical joints was developed for the effector's front section (Fig. 2b:4). The hinged tool center point (TCP) sub-system (iv) integrates an orientation between 90° and 45° relative to robot axis 6. It was composed of a steel tubular profile (200 mm-long with a 10-mm diameter) of adjustable orientation. The end-effector tool direction was defined parallel to the tube's axis (Fig. 2a:4a, 2b:4a).

Integrated fiber tension measurement (iii) was implemented through an in-line yarn tension sensor (radial strain gauge; type: Tensometric M-1191-KA^55^; nominal load: 40 N; custom-fitted with a bearing axle). The unidirectional load cell was custom configured to register forces up to 600 N by controlling the wrapping angle on the sensor roller. The device was mounted on the end-effector metal frame (Fig. 2b:7). The sensor measures the radial force acting on a ball-bearing roller. The measured values are amplified and the 16-bit integer output values calibrated to our application needs (forces of 600 N) by adjusting the offset and slope of a linear equation. The sensor was positioned as close as possible to the TCP (Figs. 1b:5a and 2b:7), ensuring accurate tension measurement before the fiber-deposition point. After force measurement, the analog signal is passed to a signal integration unit and via Ethernet to the Central Programmable Logic Controller (PLC) and the robot controller (see the Cyber Physical System Integration section).

The automatically controlled pump (see Table 3) is connected to the impregnation cartridges^50^ and supports two operation modes:

**Table 3.** Peristaltic Pump Operation, Inputs/Outputs

Inputs	Type	Description
Run	Boolean	Pump is running
Fault	Boolean	Pump has a fault
Warning	Boolean	Pump has a warning
Ready	Boolean	Pump is ready to be controlled
FrReached	Boolean	Set frequency has been reached
Outputs	Type	Description
CW	Boolean	Run pump clockwise
CCW	Boolean	Run pump counterclockwise
FastStop	Boolean	Emergency stop
VoltageLock	Boolean	Enabling/disabling the DC link voltage on the inverter
FlowRate	Double (32 bits)	Flowrate target

CCW, counter clockwise; CW, clockwise.

- Manual operation: for testing, calibration, filling, and evacuating the epoxy resin
- Automatic operation: for CNC control through the pump PLC

### Cyber-physical RCFW

We define CPRCFW by utilizing the communication and control criteria set out by Monostori *et al.*^23,56^ and Cardin *et al.*,^26^ adapting our development to the prefabrication of tubular fiber lattices for construction applications. The CPRCFW needs to integrate precision, speed, repeatability, and programmable logic control inherent to industrial robots, with the constraints imposed by an anisotropic G/CFRP material system.

#### Kinematic system

An industrial robot on a track was used as a starting point for the CPRCFW application. The KR420 fulfills the application's requirements regarding process forces from applied tension and robot end-effector weight (∼10 kg). In the present implementation, two physically independent winding tools are digitally synchronized through the kinematic system. This now contains individually programmable rotational positioners at each end (Fig. 1a), suppressing a previously utilized metal synchronization axle weighing 50 kg/m, impractical for extended setup lengths. Each winding tool now only weighs 75 kg. The weight of the system is thus reduced from ∼650 to 150 kg, whereas the setup's scalability is vastly improved. In combination with the existing 10-m track, our system can cover the complete winding range of 1 to 10 m without any change in tooling.

#### Offline robot programming

**FIG. 3.**
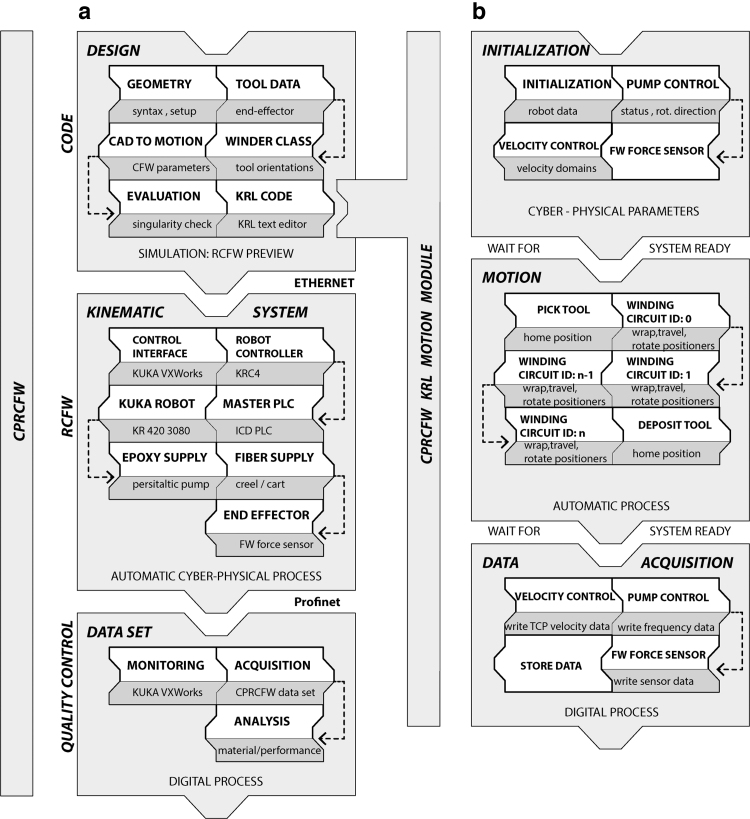
Cyber-physical integration and robot control: **(a)** The Integration of the cyber-physical components of the fabrication system; **(b)** Robot code organization, structure of the CPRCFW control module.

The robot programming model consisted of an adaptive simulation built in Grasshopper^57^ and Rhinoceros.^58^ The model contains multiple custom-built algorithms for robot motion-planning (Fig. 3a). The winding process is simulated with an inverse-kinematics solver from “Virtual Robot,” a plugin developed at ICD. The simulation provides a geometric representation of the CPS components, including the fiber syntaxes to be wound. The simulation also integrates physical system components such as the hardware and the material systems, including the robotic end-effector, and peristaltic pump. All cyber-physical components of the system are integrated through KUKA WorkVisual.^59^ The robotic motion and the functionality of the CPRCFW system are programmed through a custom-developed control algorithm developed around a” Winder” class in Python^60^ that manages all fabrication-related information.

#### Cyber-physical system integration

The components of the nine-axis kinematic system are integrated by the primary PLC (Figs. 1 and 3a). The linear track and two positioners are controlled as external motion axes. The synchronization of the rotation and velocity of the two positioners was realized by using a primary-secondary control configuration where the offline-programmed fabrication module supplies the target positions and velocities and the robot controller calculates the actual position and velocities for the entire kinematic system. Communication between the CAD environment (offline system) and the robot controller (online system) is realized through Ethernet (Fig. 3a). During the execution, the robot is placed in automatic mode.

The primary PLC receives the analog signal coming from the tension sensor amplifier and three signals from and to the peristaltic pump:

- a digital status signal;
- a first analog signal to the peristaltic pump, denoting the required revolutions per minute (RPM) value to be achieved;
- a second analog signal from the pump, denoting the actual RPM value achieved.

These signals are first passed to a unit for integrating digital/analog sensors and actuators (BEC Box,^61^ Fig. 1b:12) and then to the master PLC and robot controller through Ethernet by using the PROFINET^62^ protocol.

#### CPRCFW: real-time fiber tension and fiber impregnation control

In previous applications,^13,17,18^ the industrial robot executed a preprogrammed motion path with no feedback from the material system. To enhance those fabrication methods, we have introduced sensor-guided motion features complementary to the geometry-based motion planning methods described by Bodea *et al*.^35^ The sensor-guided motion relies on force-feedback from sensors described in the System for Fiber Guiding, Tension Measurement, and Fiber Impregnation section. The implemented feedback loops are:

- Negative feedback—between measured fiber tension and actual robot TCP-velocity and
- Positive feedback—between actual robot TCP-velocity and pump frequency.

Loop (i) is an example of negative feedback. The system maps the amplified 16-bit integer value from the force sensor invers-proportionately to a target velocity range (i.e., 0–250 mm/s). A force value reading above 600 N (∼60 kg), experimentally evaluated as maximum allowable, results in an immediate stop of the robot and the notification of the operator. Values below 600 N are linearly mapped to velocities between 1 and 250 mm/s.

Crucially, because linear acceleration means increased tension, as a result of this negative feedback loop the tension-velocity system reaches equilibrium. The linear mapping (see Table 4) resulted in smooth winding operations, where correct functioning of the system leads to constant robot velocity averaging 145 mm/s for GF and 102 mm/s for CF (see Table 5).

**Table 4.** Automatic Control of the Peristaltic Pump

Inputs	Unit	Value	Description
Velocity variables GF			
rVelLimitLow	m/s	0.06	Robot velocity lower limit
rVelLimitHigh	m/s	0.12	Robot velocity upper limit
rVelLimitMax	m/s	0.25	Robot velocity maximum allowed
Resin system flow rate GF			
rFLowAverage	g/s	2.00	Target value for average flow rate
rFlowMax	g/s	5.00	Target value for maximum flow rate
xEnablePumpCtrl	Boolean	1	Triggers automatic control of the pump
Transfer function between TCP velocity and pump flow rate			
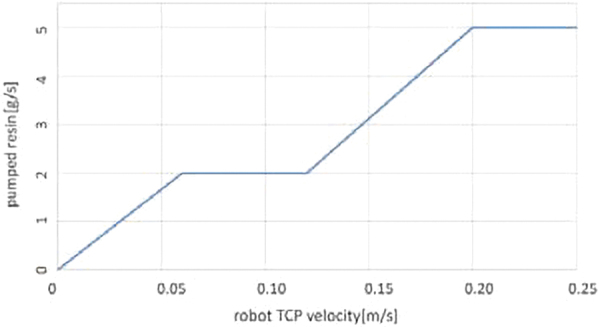			

TCP, tool center point.

**Table 5.** Material Layup of the Demonstrator

Layup	Iterations	Syntax	Fiber material:GF/CF	Syntax duration (s)	Average robot velocity (m/s)	Fiber path length (m)	Robot path length (m)
GF layup							
1	1	GF_Scaffold_1	GF	3084	0.142	430	438
3	1	GF_Body_1	GF	2775	0.147	400	408
4	1	GF_Body_2	GF	5567	0.148	809	824
CF layup							
5	1	CF_Reinforcement_1	CF	3753	0.126	438	473
6	1	CF_Boundary_1	CF	1177	0.090	45	106
7	1	CF_Boundary_2	CF	1065	0.091	40	94

From a given robot velocity and a targeted fiber/volume ratio, mass, and fiber length of a composite component we calculate a target average flow rate(F)^50^ that the pump should maintain through linear regression of observation data. The desired pump frequency(f) can then be calculated (1), utilizing the F (parametrically linked to the actual robot TCP-velocity) and the slope of the regression line(s). The pump frequency is the variable that the robot controller sends to the pump. Loop (ii) is an example of positive feedback. Robot velocity is mapped to pump frequency.

(1) $$f=\frac{F}{k};$$

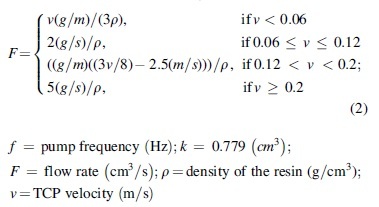


The CPRCFW motion planning is programmed by a “Winder” class (Fig. 3a). Once visual and procedural winding viability checks are completed the motion is simulated, and the CPRCFW module is automatically generated and passed to the robot controller. A CPRCFW module written in the KUKA Robot Language (KRL) controls the entire RCFW process. The composition of the control module is represented in Figure 3b.

Practically, the force sensor amplifier box and the peristaltic pump PLCs were connected to the I/O modules of the BEC Box. The Factory PLC (Fig. 1a:2) maps all signals to the robot controller, rendering them available for direct programming. The variables denoting the adjusted fiber tension (AFT), adjusted robot velocity (ARV), and adjusted pump frequency are set in two custom data modules defined in the robot controller:

- The Velocity Control(.dat) data module—contains initialization of the velocity control parameters
- The Pump Control(.dat) data module—contains initialization of the pump control parameters

These data modules are user-accessible and were configured with values specific to either GF or CF. Corresponding subprograms related to velocity and pump control are also defined in the robot controller.

- The Velocity Control(.sub) subprogram—performs all conversions from sensor output data to robot velocity and contains the velocity control logic describing the negative feedback loop described earlier
- The Pump Control(.sub) subprogram—performs all conversions from robot velocity to pump frequency and contains the pump control logic describing the positive feedback loop described earlier

In addition, a subprogram was written to manage the fabrication data acquisition:

- The Dataset(.sub) subprogram—opens a data file and creates a multidimensional array for a dataset that will contain fabrication time stamps, robot actual velocity values, tension sensor values, and pump RPM values. Two custom functions are also defined:
- AcquireData is defined inside Dataset.sub. It creates data arrays for the variables enumerated earlier. This function is called inside the winding module during the initialization steps of the executable winding module
- WriteData is also defined inside Dataset.sub. This function is called inside the winding module once all winding points have been wound. The function writes the stored data in a fabrication dataset (.txt) file inside the robot controller

The structure of the control modules mentioned earlier contributes to the system's modularity. The control code itself is modular, allowing the instructions to be efficiently regenerated on the fly. The main section integrates the cyber-physical components: the peristaltic pump and force sensor and initiates the custom velocity control loop. Each winding path is encapsulated in a fold that alternates “wrapping” and “travel” instructions, individually callable through a unique identifier. After executing the motion instructions, the components of the system are disabled and a fabrication dataset file is written as explained earlier. The CPRCFW system composition and control module are diagrammatically described in Figure 3.

## Results

**FIG. 4.**
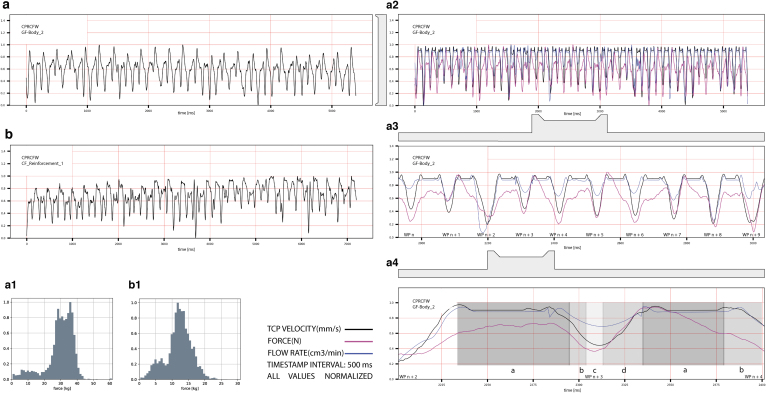
CPRCFW fabrication data set: **(a)** fiber pretension data set Syntax GF-Body02; **(a1)** distribution of tension values for GF; **(b)** fiber pretension data set Syntax CF_Reinforcement_1; **(b1)** distribution of tension values for CF; **(a2)** Complete GF dataset Syntax GF-Body02, showing correlation of fiber pretension, TCP velocity, and epoxy pump flow rate; **(a3)** Zoom-in winding points *n* + 2 to *n* + 4; **(a4)** Analysis visualization of CPRCFW process parameters for winding point *n* + 3: a—constant pretension sequence, b—decreasing tension sequence, c—low winding pretension at winding pin WP *n* + 3; figure key: TCP velocity: *black line*, force (kg): magenta line, flow rate: *blue line*, timestamp interval: 500 ms, all values normalized.

**FIG. 5.**
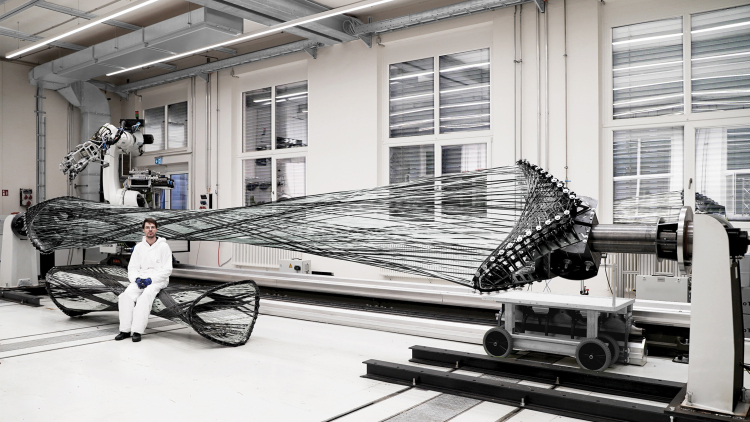
CPRCFW G/CFRP proof-of-concept (on fabrication setup) next to RCFW G/CFRP component fabricated for the BUGA Fibre Pavilion (2019). Cyber-physical fabrication methods enable an enhanced fabrication workflow, resulting in enlarged design and solution spaces. RCFW, robotic coreless filament winding.

The CPRCFW methods were verified through the data acquisition, analysis (Fig. 4), and fabrication of a tubular hyperboloid fiber structure (Fig. 5). The design of the fiber layup utilized methods described in Zechmeister *et al.*^20^ upscaled and adapted to the new fabrication and material system specifications.

The connectivity of the fiber strands is encoded in a polyline. Fiber rovings are initially wound straight and subsequently deform into a fiber lattice^63^ that approximates an anticlastic surface. The fiber layup is composed of individually tailored *fiber syntax*es.^20^ A CFW syntax is an ordered list of winding pin indices that describes how spatially arranged winding pins are connected through winding. They (Table 5) fulfill either a form-giving (GF) or reinforcement (CF) functions. The geometric instances of a fiber syntax are the primary input for the RCFW robot motion algorithm. In the CPRCFW process, epoxy-resin-impregnated fiber bundles are continuously spanned between the winding tools described in Bodea *et al.*,^19^ with the crucial difference that, for the presented application, the tools are digitally synchronized (see the Offline Robot Programming section).

The fiber layup was composed of six syntaxes (Table 5). A form-giving fiber support surface was initially wound, totaling more than 7.2 km of GF. The CF reinforcement was wound next. In total, more than 3 km of CF fibers were wound in three different syntaxes. The resulting composite was cured for 8 h at 100–120°C, resulting in a self-supporting fibrous artefact 9 m long and weighing 45 kg at an average weight of 2.3 kg/m^2^.

## Evaluation

However, it is important to note that this research did not aim at creating load-bearing construction components, which require significantly more material and suitable structural evaluation, but at demonstrating a new fabrication method. During CPRCFW, the robot executes self-similar motion sequences. Variation is induced by geometric parameters, depending on the syntax and position of the winding pins. A fabrication dataset was recorded and preprocessed for every syntax wound, following steps detailed in Appendix A1. It contained:

- robot velocity at TCP;
- fiber tension;
- pump frequency values.

Figure 4 exemplifies a normalized data sample of syntax GF-Body_2 and CF_Reinforcement_1 (Table 5). Although the robot executes spanning winding motions, the pretension value is relatively stable (Fig. 4a:4a). Subsequently, due to a reorientation sequence before/after the fiber wrapping motion, two acceleration spikes are observed (Fig. 4a:4a). Although fluctuating, the tension values remain relatively stable with tension peaks/drops and velocity directly correlated. The fiber wrapping motion exhibits a deceleration (Fig. 4a:4b) followed by lower fiber tension while wrapping the fiber (Fig. 4a:4c). During the wrapping sequence, the fiber tension stays relatively constant at half its spanning value—30 kg. During the wrapping sequence (Fig. 4a:4c), the robot increases the tension and the TCP passes around the winding pin and the cycle repeats (Fig. 4a, b).

Initial CPRCFW tests on a 4 m setup indicated fiber tension levels of up to 10 kg; for our demonstrator, average tension levels reached 30–40 kg, with peak values up to 60 kg (Fig. 4a:1, b:1). Tension values higher than 600 kN (∼60 kg) occurred due to faults in the winding effector (tangled fibers), or during missed hooking sequence. However, all mechanical faults were remedied and the system functioned robustly during the demonstrator's production phase. A tension fluctuation (Fig. 4a:4) of around 20% was experienced. This is unsurprising given the freeform geometry of the demonstrator. Due to a higher tension setting applied in the mechanical tensioning system, for CF the fiber tension levels recorded were higher than those experienced for GF (Fig. 4b:1, a:1). The characteristic tension values for different materials are presented in Table 6. The normalized datasets for GF and CF utilized for visualizing the force distributions are presented in Figure 4a:1 and 4b:1.

**Table 6.** Characteristic Tension Values for the Fabrication of the Large-Scale Demonstrator

Syntax	Material	During hooking	During traveling
Body	GF	8 kg	19 kg
Reinforcement	CF	10 kg	28 kg
Corner	CF	7 kg	32 kg

## Discussion

The initial phase of RCFW presented in Bodea *et al.* demonstrated that the technology could be applied to industrialized prefabrication. However, the fabrication data analysis suggested that the upscaling and productivity potential of the application could be further explored.^35^

An initial effect of the upscale are significantly higher process forces—30–40 kg versus 10 kg—in previous applications. Consequently, the tooling required added engineering robustness precision. The robot end-effector was designed to function at velocities up to 500 mm/s regardless of orientation.

During preparation and calibration, the end-effector's tensioning mechanism did not perform robustly. Frequent automatic interruptions were caused by abrupt drops in fiber tension, which prevented the system from reaching equilibrium in correlation with TCP velocity. It was determined that the tensioning subsystem integrated in the end-effector was generating increased fiber tension due to complex routing of the individual fiber rovings (Fig. 2a). As a solution, the internal tensioning mechanism and the routing of the rovings were rationalized (Fig. 2b).

A second upscaling consequence was a 10–20% tension fluctuation, owing to the complexity of the fiber syntax. Eliminating these fluctuations through mechanical compensation means is impractical, thus a future solution would need to include active fiber tension control for each fiber bobbin.

A third upscaling consequence was longer cycle times. The dynamically controlled robot velocity introduces unpredictability in the process. However, the presented development enables a comprehensive simulation of the fabrication process based exclusively on material properties, previously impossible due to a lack of fabrication data. Moreover, longer cycle times impacted the selection of a material system with a lower increase in viscosity over time and theoretically unlimited open time compared with a 5-h open time specified in Bodea *et al*.^35^ As a result of added process complexity, the majority of R&D work addressed the integration and calibration of the CPRCFW system as opposed to intensive manual labor in previous applications. As a result, better impregnation quality and more precise fiber tension and robot velocity control could be achieved even for G/CFRP elements double the previously achievable length.

Overall, these features led to a reevaluation of the role of the human-in-the-loop, decreasing the specialization demanded from technicians and robot operators. The result was a more automated AM process, where many process parameters are derived from the internal state of the system and where humans are tasked with monitoring and control. Concurrently, programming of the system became simpler and more intuitive, owing to enhanced automation and integration.

An added contribution of this research is the potential enhancement of simulation methods for RCFW, and real-time response to material system constraints registered during CPRCFW. We next present some scenarios illustrating how simulation methods and online process adjustments will inform future interdisciplinary design-engineering methods.

In a first scenario, the acquired fabrication data contribute to an expanded computational design space. The existing design process^20^ utilizes a simplified dynamic relaxation to approximate anisotropic material behavior. This method assumes that fiber tension remains constant during winding. This implies that some of the energy is dissipated through material stiffness whereas some accumulates, resulting in constantly increasing pretension. However, our fabrication dataset demonstrates that in RCFW fiber tension is variable, owing to complex geometry and robot motion. The presented dataset and processing methods can be directly utilized for a more informed dynamic relaxation simulation. Moreover, it was experimentally observed that the overall elasticity of the lattice decreases proportionally to the number of fibers wound, yet this decrease is not linear and has not yet been mathematically described owing to a lack of quantifiable fabrication data. In addition, the current dynamic relaxation model^20^ does not predict the effects of increasing or decreasing fiber tension. The resulting design method is informed by anisotropic material properties combined with reciprocal deformation of the fibrous lattice effects. Consequently, the utilization of accurate fabrication data would aid the modeling of changes in fibrous lattice elasticity and provide a quantitative basis to quantify the design and structural benefits related to this material-system property.

Building on the more accurate simulation from the first scenario, in a second scenario, the fabrication data inform an enhanced structural simulation to more accurately predict the form of the structural lattice, translating to a precise structural evaluation of the amount of pretension induced.

These scenarios allow us to conceptualize adequate post/during-fabrication-measures to respond to emerging structural or building system constraints. Postfabrication measures trigger changes in computational models to adjust material amount, impacting the fiber syntax topology and component geometry. These adjustments would affect the design of subsequent components. During-fabrication-measures include procedurally added, topological, or geometric changes in syntax layup as well as procedurally AFT with a direct impact on fiber lattice morphology during the winding process.

## Conclusion and Outlook

This article discussed the opportunities and challenges presented by CFW for the building industry. The core contributions were an increase in automation for RCFW coupled, with a significant upscale of the process. The emerging demand for more general, versatile, and reusable RCFW methods and tools was addressed by a CPRCFW method proposing an abstraction of control methods, physical and software tool-modularity, and clearer automation protocols. The methods were embodied by online sensor-informed tools for process monitoring and control.

In addition, this article exemplified the upscaling potential of the technology, resulting in larger building components. The CPRCFW application was verified through the fabrication of a 9 m long composite element consisting of a bespoke G/CFRP fiber layup tailored to the specifications of the prefabrication setup. The automated process demonstrated robustness and reliability, reflected by the fabrication dataset analysis, which showed close correlation between the fabrication parameters of robot winding velocity and fiber tension.

The importance of online controlled fabrication through sensor feedback for future more informed design-engineering-fabrication methods was discussed in several discipline-relevant development scenarios.

The realized demonstrator suggests that the linear scalability of the chosen component typology is well supported by the fabrication method. In practical terms, this translates to the capacity to cover more than 1200 m^2^ with a 14 m high structure, triple the area and double the height achieved by the BUGA Fibre Pavilion^35^ with a similar building system, utilizing our tooling, control methods, and automation protocols while incurring minimal added automation/production costs.

A wide-ranging interdisciplinary investigation aiming at disentangling design and solution space limitations, with profound implications for composite architecture applications, is underway in the context of the DFG Cluster of Excellence Integrative Computational Design and Construction.^64^ This initiative is planned to yield novel research advancements and construction-scale results within the next 2 to 3 years.

Our investigations represent incremental research toward more autonomous prefabrication environments through CPRCFW. Our goal was to expand the range of fibrous morphologies and material systems compatible with its construction application. Although currently calibrated for two types of fibers, GF and CF, we estimate that this technology is applicable to many other material systems, including those sourced from renewable resources such as composites utilizing plant-based or basalt fibers and ecologically competitive resin systems.

Presently, several technologies involving robotic AM and bespoke CPS including CFW find themselves in a knowledge-transfer relationship with the construction industry. However, efficient robotized production still requires incremental advancement in material and building system-aware communication and control. This constitutes a proving ground for technologies, such as CPRCFW, trying to solve the challenges of automation and AM in construction.

## Appendix

### Appendix 1. Data Preprocessing Steps

The fabrication dataset was preprocessed according to the following steps:

- Prolonged periods of downtime—when the robot velocity was 0.00, they were filtered out. They signify a mechanical/hardware or software fault that had to be remedied
- The tension sensor values are amplified by direct multiplication with a sensitivity coefficient to reflect the physical orientation sensor
- The tension sensor values are subsequently remapped to the metric system kg unit of measurement
- Peristaltic pump values are converted from revolutions per minute to a flow rate to better reflect epoxy resin consumption
- Tension sensor data, robot velocity at tool center point data, and flow rate data are then normalized by remapping the values from their respective units of measurement and domains to a [0,1] domain
- The digital filter Savitzky–Golay^65^ (data range 51, polynomial order 2) is applied to the data, with the purpose of smoothing and increasing the precision of the data points without affecting the signal tendency
